# Propolis Induces AQP3 Expression: A Possible Way of Action in Wound Healing

**DOI:** 10.3390/molecules24081544

**Published:** 2019-04-19

**Authors:** Simona Martinotti, Giorgia Pellavio, Umberto Laforenza, Elia Ranzato

**Affiliations:** 1 DiSIT—Dipartimento di Scienze e Innovazione Tecnologica, University of Piemonte Orientale, Viale Teresa Michel 11, 15121 Alessandria, Italy; 2 Department of Molecular Medicine, Human Physiology Unit, University of Pavia, 27100 Pavia, Italy; giorgia.pellavio01@universitadipavia.it (G.P.); lumberto@unipv.it (U.L.); 3 DiSIT—Dipartimento di Scienze e Innovazione Tecnologica, University of Piemonte Orientale, Piazza Sant’Eusebio 5, 13100 Vercelli, Italy; elia.ranzato@uniupo.it

**Keywords:** aquaporin-3, ROS, migration, propolis, wound repair

## Abstract

Propolis is the generic name of a complex of resinous compound collected by honeybees and it has been utilized for many years in folk medicine. As other products generated by honeybees (such as royal jelly, pollen, honey), propolis has great therapeutic properties, but very little scientific information is available. Therefore, this study was aimed at exploring the potential wound healing properties of propolis. To that end, we utilized an in vitro scratch wound healing model consisting of human immortalized keratinocytes. Our scratch wound data clearly demonstrated that propolis induced a pronounced increase in the wound repair abilities of keratinocytes. A cell migration assay showed that propolis stimulated keratinocytes to close the wound. We revealed the role of H_2_O_2_ as the main mediator of propolis regenerative properties. We showed that this extracellularly released H_2_O_2_ could pass across the plasma membrane through a specific aquaporin (i.e., AQP3) modulating intracellular responses. The data offer a biological characterization of propolis positive effects suggesting that propolis could also be utilized in wound treatment within clinical settings.

## 1. Introduction

Propolis is the generic name indicating the complex of resinous substances gathered by honeybees from some parts of plants, buds, and exudates in the North Temperate Zone, from the Tropic of Cancer to the Arctic Circle. The main sources of propolis are poplar, willow, birch, elm, alder, beech, conifer, and horse-chestnut trees [1]. Propolis has been used extensively in folk medicine for many years, and there is substantial evidence indicating that propolis has antiseptic, antifungal, antibacterial, antiviral, anti-inflammatory and antioxidant properties [1,2,3].

As other products obtained from honeybees (such as royal jelly, honey, pollen), propolis shows significant therapeutic values, being utilized since antiquity in various parts of the world, but very little scientific information is available [1].

Given the countless interest in developing new wound healing products and characterizing their mechanism of action, this study was planned to explore in vitro the potential wound closure effects of propolis.

We used an in vitro scratch wound healing model consisting of human immortalized keratinocytes, HaCaT cells [4]. In vitro tests are now broadly accepted and utilized in research because of ethical aims and their helpfulness in bioactive-guided active complexes fractionation and determination [5].

Our observations showed that propolis remarkably boosted wound closure. Then we investigated the interactions between extracellularly produced H_2_O_2_ and aquaporin-3 (AQP3). AQP3 is an aquaporin water channel family member, and it is a water- and glycerol-transporting protein with an important role in several cellular functions [6]. AQP3-facilitated water transport plays a pivotal role in cell migration, accelerating the healing of cutaneous wounds. Moreover, AQP3-facilitated glycerol transport is involved not only in skin hydration and elasticity, but also in cell proliferation [7].

Previous studies have already revealed the role of AQP3 expression in wound healing as well as its involvement in mediating H_2_O_2_ signalling [8].

Our data suggested that reactive oxygen species (ROS) generated by propolis exposure could diffuse across the plasma membrane through AQP3 modulating intracellular responses.

Taken together, these data suggest the pharmaceutical implementation of propolis as an adjuvant in wound healing treatments.

## 2. Results

### 2.1. Cell Proliferation and Metabolism

Propolis cytotoxicity tests were performed on HaCaT cells to optimize the amounts for in vitro scratch wound studies. 

Calcein-AM assay data showed extremely low cytotoxicity levels for the propolis sample (Table 1). Based on these data, in subsequent experiments propolis concentrations below EC_05_ were used.

### 2.2. Scratch Wound Repair of HaCaT in the Presence of Propolis

Confluent monolayers of HaCaT cells were scratch wounded as described in the Materials and Methods section and then allowed to re-epithelialize for 24 h at 37 °C in the presence or absence of propolis at the specified concentrations. 

One series of samples, utilized as positive controls, were exposed to 20% (*v*/*v*) of a platelet lysate (PL), achieved from blood samples [4,9]. PL is broadly used in wound regeneration, and we previously demonstrated that PL promotes scratch wound healing in HaCaT cells [4]. 

**Figure 1:**
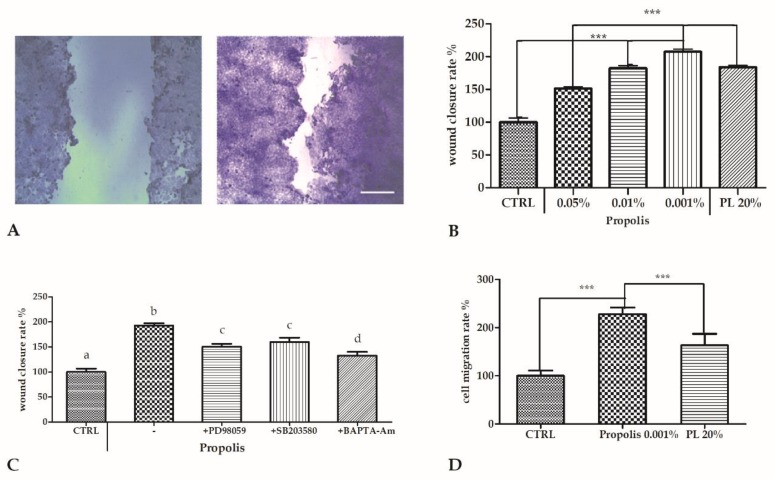
Scratch wound healing of HaCaT confluent monolayers. Cells were cultured in 12-well plates and mechanically scratched with a sterile 0.1–10 μL pipette tip, and then allowed to re-epithelialize for 24 h at 37 °C in the presence of different propolis concentrations (*v*/*v*). One sample was exposed to 20% platelet lysate (PL) as a positive control. (**A**) Micrographs of scratch wounded HaCaT monolayers incubated under control conditions (CTRL, left) or in the presence of 0.001% propolis (right) and then stained with blue toluidine and observed 24 h after wounding. Scale bar, 200 μm. (**B**) Measurements of wound closure expressed as the difference between wound width at 0 and 24 h. Bars represent mean ± SD of two independent experiments, each with *n* = 25. The mean of the control was set to 100. The statistical difference was determined by a one-way ANOVA followed by Bonferroni’s Multiple Comparison Test (*** *p* < 0.001). (**C**) Effect of different inhibitors on propolis-induced scratch wound repair of HaCaT monolayers. Data were recorded 24 h after scratch wound healing of cells exposed to 0.001% propolis, in the presence or absence of various inhibitors. The bars represent mean ± SD of percent wound closure inhibitions recorded in two independent experiments, each with *n* = 20 and the statistical difference was determined by a one-way ANOVA followed by Bonferroni’s Multiple Comparison Test (*** *p* < 0.001). **D.** Effect of 0.001% propolis and of 20% PL as the positive control, on HaCaT cell migration evaluated by transwell migration assay (see Methods). Data are mean ± SD (*n* = 5) of cell migration rate (see text) expressed as percent variation with respect to the control. Statistics as in B and C.

Cells exposed to propolis showed a significantly higher wound closure rate with respect to the control. The strongest effect was observed with 0.001% of propolis, more pronounced from that induced by PL (Figure 1A,B).

Then, to explore the mechanism of action of propolis on wound closure, we performed a new series of scratch wound assay experiments in the presence of some well-characterized inhibitors, such as PD98059 (an ERK inhibitor, 10 µM), SB203580 (a p38 inhibitor, 20 µM), and the cell-permeant calcium chelator BAPTA-AM (30 µM).

To that end, confluent cells were scratched with or without each inhibitor, with or without 0.001% propolis, and the wound closure rate was recorded at 24 h post-wounding.

The increase of wound closure rate induced by propolis exposure was inhibited to various extents in this order: SB203580 > PD98059 > BAPTA-AM (Figure 1C).

The vehicle alone (0.1% DMSO) did not influence wound closure, in either the presence or absence of propolis (data not shown).

### 2.3. Propolis Chemoattractant Effect

To evaluate whether propolis influenced cell migration rates, we performed a chemotaxis assay using 0.001% propolis (*v*/*v*), the most effective concentration in inducing wound closure. The results showed that in the presence of propolis, the number of migrating cells significantly increased with respect to the control (*p* < 0.01). Propolis exposure also produced an effect stronger than induced by 20% PL (Figure 1D).

### 2.4. Aquaporins (AQPs) Expression upon Propolis Exposure

Aquaporins (AQPs) are integral membrane proteins; they act as channels in the water transfer across the plasma membrane, playing a central role in skin hydration [8,9]. Therefore, we decided to quantify the basal expression of some AQPs and the variation after propolis exposure, by means of qPCR data.

**Figure 2:**
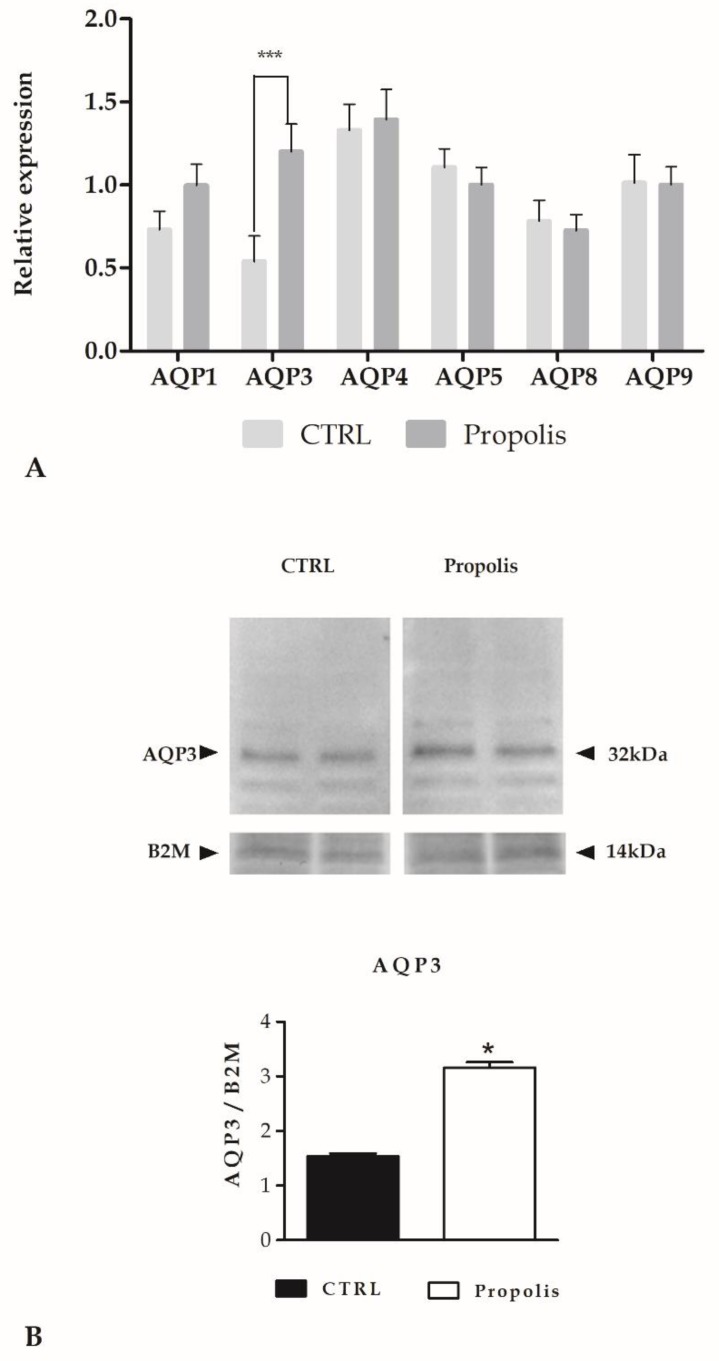
Aquaporins (AQPs) expression. (**A**) Expression of AQPs genes in HaCaT cells treated with propolis. The mRNA quantity of several AQPs was determined by qRT-PCR and is expressed as mean relative expression ± SD (*n*=3). The statistical difference was determined by a two-way ANOVA followed by a Bonferroni post-test (*** *p* < 0.001). (**B)** Aquaporin-3 (AQP3) protein expression in HaCaT cells after propolis exposure. Blots representative of two were shown. Lanes were loaded with 30 μg of proteins, probed with anti-AQP3 rabbit polyclonal antibodies and processed as described in the Materials and Methods section. The same blots were stripped and re-probed with anti-beta-2-microglobulin (B2M) polyclonal antibody, as housekeeping. A major band of about 32 kDa was observed (* *p* < 0.001, *t*-test).

In Figure 2A, we analysed the basal expression of AQP-1, -3, -4, -5, -8 and -9 in keratinocytes, but only the expression of AQP3 was significantly enhanced after propolis exposure, as confirmed also by western blotting analysis (Figure 2B).

Together with other studies, we demonstrated that AQP3 was able to mediate the transport of H_2_O_2_ in different tissues and conditions [7,8,10,11], therefore, we decided to study the role of AQP3 upon propolis exposure as a mediator of H_2_O_2_ signalling that could trigger intracellular responses.

**Figure 3:**
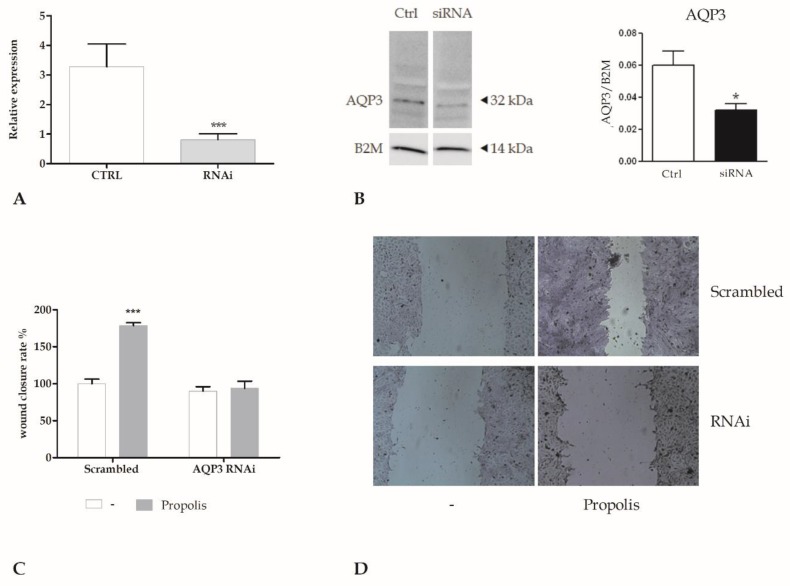
The genetic silencing of AQP3 abolishes the wound closure induced by propolis. (**A**). Expression of AQP3 gene in HaCaT cells after RNA interference (RNAi). The mRNA quantity of AQP3 was calculated by qRT-PCR and is expressed as mean relative expression ± SD (*n* = 3, * *p* < 0.001, *t*-test). (**B**) AQP3 protein expression in HaCaT cells (CTRL) after AQP3 RNAi (RNAi). Blots representative of three were shown. Lanes were loaded with 30 μg of proteins, then probed with anti-AQP3 rabbit polyclonal antibody and managed as described in the Materials and Methods. The same blots were stripped and re-probed with anti-beta-2-microglobulin (B2M) antibody, as housekeeping (* *p* < 0.001, t-test). (**C**) Measurements of wound closure in scrambled cells or in cells exposed to RNAi for AQP3 (AQP3 RNAi), in the presence or not of 0.001% propolis, calculated as the difference between wound width at 0 and 24 h. Bars show mean ± SD of two independent experiments, each with *n* = 25. The mean of the control was set to 100 (*** *p* < 0.001, Bonferroni post-test). (**D**) Micrographs of scratch wounded HaCaT monolayers. Scrambled cells or cells exposed to RNAi for AQP3 (AQP3 RNAi) were incubated in the presence of 0.001% propolis and then stained with blue toluidine and observed 24 h after wounding.

To assess the role played by AQP3, we performed silencing of AQP3 with specific siRNA oligonucleotides by using the N-ter Nanoparticle siRNA Transfection System (Figure 3A,B). In order to assess the role of AQP3 in propolis-boosted wound closure, we performed the scratch wound assay (Figure 3C,D) in silenced keratinocytes. We could not observe any significant variation in the rate of wound closure, confirming the pivotal role of AQP3 in mediating wound closure induced by propolis.

### 2.5. Role of AQP3 in H_2_O_2_ Influx

**Figure 4:**
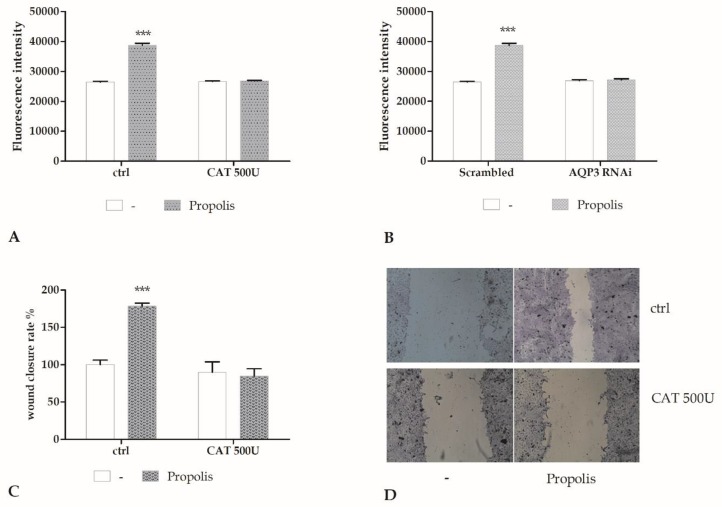
Propolis-induced wound closure requires hydrogen peroxide. (**A**) Fluorescence values, corresponding to H_2_O_2_ increasing concentrations were recorded at 10 min in control cells (ctrl), or in cells incubated with catalase (500 U, CAT, 30 min preincubation), in the presence or not of 0.001% propolis (*** *p* < 0.001, Bonferroni post-test). Data are presented as means ± SD of rhodamine 123 fluorescence expressed in arbitrary units; *n* = 16 microplate wells from two different experiments. (**B**). Fluorescence values recorded at 10 min in scrambled cells or in cells with RNAi for AQP3 (AQP3 RNAi), in the presence or not of 0.001% propolis (*** *p* < 0.001, Bonferroni post-test). Data are presented as means ± SD of rhodamine 123 fluorescence expressed in arbitrary units; *n* = 16 microplate wells from two different experiments. (**C**) Measurements of wound closure in control (ctrl) cells or in cells exposed to CAT 500 U, in the presence or not of 0.001% propolis, expressed as the difference between wound width at 0 and 24 h. Bars represent mean ± SD of two independent experiments, each with *n* = 25. The mean of the control was set to 100 (*** *p* < 0.001, Bonferroni post-test). (**D**) Micrographs of scratch wound. Control (ctrl) cells or cells exposed to CAT 500 U were incubated in the presence of 0.001% propolis and then stained with blue toluidine and observed 24 h after wounding.

Propolis can induce, in cell cultures, H_2_O_2_ production [12]. By using the xylenol orange assay, we evaluated the H_2_O_2_ production induced by propolis around 0.5 H_2_O_2_ ng/mL. Therefore, we hypothesized that, once released into the extracellular milieu, H_2_O_2_ would distribute across the plasma membrane to increase intracellular ROS concentration. To demonstrate that, we measured the intracellular ROS increase by recording the DHR-123 loaded HaCaT cells fluorescence in a microplate reader. Figure 4A,B show the induction of a sustained rise in ROS levels in keratinocytes after propolis exposure, while the catalase (CAT a scavenger of H_2_O_2_) and RNAi for AQP3 abrogated this effect.

We then assessed whether the presence of exogenous CAT, which scavenges H_2_O_2_, reduced the propolis effect on wound closure. Pre-treatment of cells with CAT abrogated the wound closure induced by propolis (Figure 4C,D) as well as what was already observed in Figure 3B with RNAi for AQP3.

## 3. Discussion

Propolis has been empirically used for centuries and it has several biological applications including acceleration of regeneration processes [1], immunomodulatory [12], antimicrobial [13], antioxidant, analgesic [14], and anti-inflammatory effects [15].

More than 300 substances have been identified as chemical components of propolis. The proportions of these compounds in propolis depend on the local flora [16,17]. The levels of chemical compounds in different kinds of propolis extracts, such as ethanolic, aqueous-ethanolic and aqueous glycolic has already been compared [18]. The ethanolic preparation was composed of a great amount of resveratrol, chrysin and caffeic acid compared to others. Therefore, we decided to test the biological effects of ethanolic extract of propolis.

Among the main components of propolis we had phenolic compounds (flavonoids, aromatic acids, and benzopyrenes), di- and tri-terpenes, essential oils, aromatic acids and esters, aldehydes, ketones, and phenylpropanoids (i.e., caffeic acid). Flavonoids and phenolic acids [19] are the components directly related to antibacterial, anti-inflammatory and wound healing properties, but the biological potential of propolis is a result of synergistic interactions between its components [16], because the isolated compounds do not induce the same effects as the total extract [20,21].

Propolis has not yet been tested on wound mechanisms and we decided to perform a battery of experiments in order to get better insight on its effects on a wound healing model.

The propolis treatment displayed low cytotoxicity on HaCaT cells, as already proposed in animal experiments [22,23]. Therefore, these observations suggest that propolis can be considered as a safe substance for external applications not only for healthy skin, but also as a product for dressing wounds and burns.

Our scratch wound data clearly demonstrated that propolis induced a pronounced increase in wound repair abilities of keratinocytes. We also compared this effect to the stimulation induced by a PL [4], that is usually applied in clinical practice [24]. Our data also revealed that propolis was able to induce the wound closure on HaCaT cells to a different extent. At 0.01%, propolis induced an effect similar to that exerted by PL and at 0.001% it was able to stimulate the wound closure to an extent greater than that obtained with PL. 

Moreover, the cell migration assay showed that propolis stimulated the ability of keratinocytes to close the wound. In particular, we observed propolis inducing strong chemotaxis effects compared to that obtained with 20% PL. 

Furthermore, in order to recognize the basic mechanisms of this propolis-induced exploit, we utilized an inhibitor battery of main cell signaling pathways, known to be directly involved in tissue regeneration [4]. BAPTA-AM resulted as the most effective inhibitor of scratch wound closure assay, confirming the essential role of intracellular calcium, as previously pointed out on these cells [9], while the use of SB203580 and PD98059 showed the minor contribution of p38 and ERK1/2 pathways. 

Propolis is an anti-oxidant, however, it may also act as a pro-oxidant and can induce oxidative stress under certain circumstances [25]. Tsai et al. already demonstrated that transition metal ions were required for propolis-induced oxidative stress and reactive oxygen species such as H_2_O_2_ were generated in cell culture [25]. Phenolic compounds present in propolis such as galangin, chrysin, and pinocembrin are temporary electron carriers of redox reactions series, in which electrons from ferrous ions are relayed to oxygen molecules generating superoxide, after which H_2_O_2_ is generated [25]. It should be noted that the H_2_O_2_ production induced by propolis was very low and at these concentrations, H_2_O_2_ could show a positive role as major redox metabolite operative in redox sensing, signaling and redox regulation. In this light, we could consider the higher effect of 0.001%, with a mixture of low pro-oxidative effect and moderate anti-oxidant action. In our study, we confirmed the involvement of H_2_O_2_ as the main mediator of propolis wound closure effects on HaCaT cells, a spontaneously immortalized human keratinocyte line.

We confirmed that this extracellularly released H_2_O_2_ could pass across the membrane through a specific aquaporin (i.e., AQP3). AQP3 has already been demonstrated to have a fundamental role in the hydration of mammalian skin as well as in keratinocyte differentiation and growth [26,27]. 

The role of H_2_O_2_ released by propolis exposure is supported by the inhibition in a dose-dependent manner by CAT, an H_2_O_2_ scavenger. The use of generic fluorescent H_2_O_2_ indicator, such as DHR-123, revealed ROS production within the cytoplasm. Genetic silencing of AQP3 with a selective RNAi prevented the increase in intracellular ROS levels and abrogated the wound closure response to propolis. These data, therefore, strongly suggested that AQP3 mediated H_2_O_2_ entry into the cytoplasm.

Once in the cytoplasm, H_2_O_2_ transduced signals mediating responses to the external environment. We already revealed that honey exposure mediated in keratinocytes, through AQP3, H_2_O_2_ uptake and accumulation within the cytosol, thereby generating a signaling that could open adjacent H_2_O_2_-sensitive Ca^2+^ channels [6]. 

Such features could render the propolis very interesting for a possible synergistic interaction with other medical wound dressings, such as platelet products [4,9,28,29], and with other natural products, such as jojoba liquid wax [5] and/or honey [29].

## 4. Materials and Methods

### 4.1. Cell Culture and Reagents

All reagents were obtained from Sigma-Aldrich (St. Louis, MO, USA,), unless otherwise indicated. Crude propolis was collected from the Monferrato area (Piemonte region, Italy), and kept desiccated in a refrigerator (−20 °C) before being processed. Ethanol propolis extract was prepared by an already utilized procedure [30].

During the cell experiments, the final concentration of ethanol in the medium did not exceed 0.1% (*v*/*v*).

HaCaT cells are immortalized human skin keratinocytes that mimic several characteristics of normal epidermal keratinocytes. HaCaT cells are not invasive, and can differentiate under appropriate experimental conditions. Cells were grown at 37 °C, 5% CO_2_, in DMEM supplemented with 10% foetal bovine serum (FBS) and 1% antibiotic mixture.

### 4.2. Calcein-AM Assay

The lipophilic, nonfluorescent calcein-acetoxymethylester (calcein-AM) enters cell membranes and is then cleaved by intracellular esterases, producing hydrophilic fluorescent dye. Cells were settled overnight in 96-well plates (8000 cells/well), incubated with propolis for 24 h, washed with PBS, and then incubated for 30 min at 37 °C with a solution of 2.5 µM calcein-AM in PBS. Plates were read in a fluorescence plate reader (Infinite 200 Pro, Tecan, Wien, Austria)), by using 485-nm excitation and 535-nm emission filters. 

### 4.3. Scratch Wound Assay

Scratch wounds were performed in confluent monolayers of keratinocytes by utilizing a sterile 0.1–10 µL pipette tip. After washing away suspended cells, cells were fed with medium in the presence of different concentrations of propolis for 24 h. Thereafter, cells were fixed in 3.7% formaldehyde in PBS for 30 min, and then stained with 0.1% toluidine blue for 30 min. The width of the wound space was recorded at wounding and at the end of treatments, using an inverted microscope equipped with a digital camera (Leica Microsystems). Digitized pictures of wounds were analyzed using the NIH ImageJ software (National Institutes of Health; Bethesda, MD, USA). Wound closure rates were calculated as the difference between wound width at 0 and 24 h. 

### 4.4. Platelet Lysate Preparation

Platelet concentrates were obtained from a single volunteer donor, using platelet apheresis collection qualified for clinical use and produced by a discontinuous blood cell separator. The platelet-rich plasma was automatically separated from other blood components by centrifugation, collected into a sterile disposable bag, and then washed and concentrated to a final density of 2 × 10^9^ cells/ml. To obtain the PL, platelet concentrates were frozen (−80 °C) and thawed (37 °C) three consecutive times, the remaining platelet bodies and debris were eliminated by centrifugation (3000× *g*, 10 min) and the supernatant was stored in aliquots at −80 °C until use [9].

### 4.5. Cell Migration Assay

A cell migration assay was performed in transwell plates (8 µm pore size, Costar, Cambridge, MA). A total of 1 × 10^5^ cells per well were seeded in the upper filter compartment. After 24 h filters were removed and stained for 10 min with 0.5% crystal violet (145 mM NaCl, 0.5% formal saline, 50% ethanol), and then washed thrice with water. The upper filter side was scraped using a cotton swab to remove cells that had attached but not migrated. Following PBS washing of filters, the dye was eluted from cells with 33% acetic acid, and measured at 540 nm in a plate reader (Infinite 200 Pro, Tecan).

### 4.6. Quantitative Reverse Transcriptase PCR (qRT-PCR)

Cells were treated or not treated with propolis, and RNA was extracted and cDNA generated using a Transcriptor First Strand cDNA Synthesis Kit (Roche Diagnostics GmbH, Penzberg, Germany) qRT-PCR was performed using the iTaq Universal SYBR Green Supermix (Bio-Rad Laboratories, Hercules, CA, USA), and the CFX384 Real-Time PCR Detection System (Bio-Rad). Gene expression was calculated using the ΔΔCt method by means of CFX Manager™ Software (Bio-Rad).

The following qRT-PCR primer pairs were utilized:
β-actin5′-TCCCTGGAGAAGAGCTACGA-3′5′-AGCACTGTGTTGGCGTACAG-3′GADPH5′-AATCCCATCACCATCTTCCA-3′5′-TGGACTCCACGACGTACTCA-3′AQP15′-TAAGGAGAGGAAAGTTCCAG-3′5′-AAAGGCAGACATACACATAC-3′AQP35′-CTGTGTATGTGTATGTCTGC-3′5′-TTATGACCTGACTTCACTCC-3′AQP45’-GCTGTGATTCCAAACGGACTGATC-3’5′-CTGACTCCTGTTGTCCTCCACCTC-3′AQP55′-GCTGGCACTCTGCATCTTCGC-3′5′-AGGTAGAAGTAAAGGATGGCAGC-3′AQP85′- GGAGATAAGAGTATCTTGCAC-3′5′- CTTGTCATTGCCAAATTCAG-3′AQP95′-GTATTGGTAGAAACAGGAGTC-3′5′-GGACAATCAAGATGAACGTG-3′

### 4.7. RNA Interference

Cells were transfected with siRNA oligonucleotides (5 µM), or with equimolar scramble siRNA, by using the N-ter Nanoparticle siRNA Transfection System. We used commercial siRNA specific to the human AQP3 gene (MISSION siRNA). Scramble siRNA was achieved using commercial non-targeting siRNA (MISSION siRNA Universal Negative Control). RNA was extracted and cDNA generated as described above.

### 4.8. Immunoblotting

Cells were lysed in RIPA buffer (supplemented with a protease and phosphatase inhibitor cocktail) and homogenates were solubilized in Laemmli buffer [31]. 30 µg proteins were separated on precast gel electrophoresis (4–20% Mini-PROTEAN TGX Stain-Free Gels, Bio-Rad, USA) and transferred to the PVDF Membrane (Trans-Blot Turbo Transfer Pack, #1704156, Bio-Rad, USA) with Trans-Blot Turbo Transfer System (#1704150, Bio-Rad, USA). Blots were blocked for 1 h with Tris buffered saline (TBS) containing 5% non-fat dry milk and 0.1% Tween (blocking solution). The membranes were incubated overnight with the following antibodies diluted in the TBS and 0.1% Tween: Anti-AQP3 antibody produced in rabbit (SAB5200111; Sigma-Aldrich, Italy; 1:1000, dilution). RabMAb anti β-2-microglobulin antibody ([EP2978Y] ab75853; Abcam; 1: 10000 dilution). The membranes were washed and incubated for 1 h goat anti-rabbit IgG antibody, peroxidase conjugated (AP132P; Millipore) diluted 1:100,000 in blocking solution. The bands were detected with Westar Supernova western blotting detection system (CYANAGEN). Pre-stained molecular weight markers (ab116028, Abcam) were utilized to calculate the band molecular weights. Blots were acquired with the high-resolution scanner Expression 1680 Pro (Epson Corp., Long Beach, CA, USA). The densitometric analysis of the bands was determined by using the Total Lab V 1.11 computer program (Amersham) and the results were expressed as AQP3/B2M densitometric ratio.

### 4.9. Measurement of Intracellular ROS

The intracellular ROS level was assessed utilizing the fluorescent dye precursor dihydrorhodamine (DHR) 123, which was converted to fluorescent rhodamine 123 upon reaction with ROS. Cells were seeded in 96-well plates, allowed to settle overnight, and loaded for 30 min at room temperature in the dark with DHR-123 (30 µM) in a loading buffer consisting of (mM) 10 glucose, 10 Hepes, 140 NaCl, 1 MgCl_2_, 2 CaCl_2,_ and 5 KCl, pH 7.4. Cells were then washed with loading buffer and fluorescence recorded in a fluorescence microplate reader, by using 485-nm excitation and 530-nm emission filters. Data of ROS production were expressed as fluorescence arbitrary units [31].

### 4.10. Hydrogen Peroxide Assay

The formation of H_2_O_2_ induced by propolis in culture medium was determined by xylenol orange, a colorimetric assay, as previously described [8,31].

### 4.11. Statistical Analysis

Data were analyzed with the Instat software package (GraphPad Software, GraphPad Software Inc, San Diego, CA, USA). Median (EC_50_) and minimum (EC_05_) effective concentrations and their 95% confidence intervals were calculated by utilizing a downhill logistic dose-response curve. For statistical analysis we used one-way ANOVA or two-way ANOVA followed by Bonferroni’s Multiple Comparison Test. 

## Figures and Tables

**Table 1:** Values of effective concentrations, EC_05_ and EC_50_ (% *v*/*v*), derived from dose-response curves obtained for propolis on HaCaT cells at 24 h.

EC_05_	EC_50_
0.015% (0.012–0.019%)	0.048% (0.045–0.052%)

Experiments (calcein AM method) were carried out in triplicate with a minimum of 8 replicates each; 95% CI are given in parentheses (See Supplementary Materials).

## Supplementary Materials

The supplementary Materials are available online.
