# Supplementary material for: Propolis Induces AQP3 Expression: A Possible Way of Action in Wound Healing
Source: Molecules. 2019 Apr 19;24(8):1544. doi: 10.3390/molecules24081544 (PMC6515181; doi:10.3390/molecules24081544)
Supplement: Supplementary file 1 [file molecules-24-01544-s001.pdf]

EC_{05}	EC_{50}
**0.015 %**	**0.048 %**
**(0.012-0.019 %)**	**(0.045-0.052 %)**

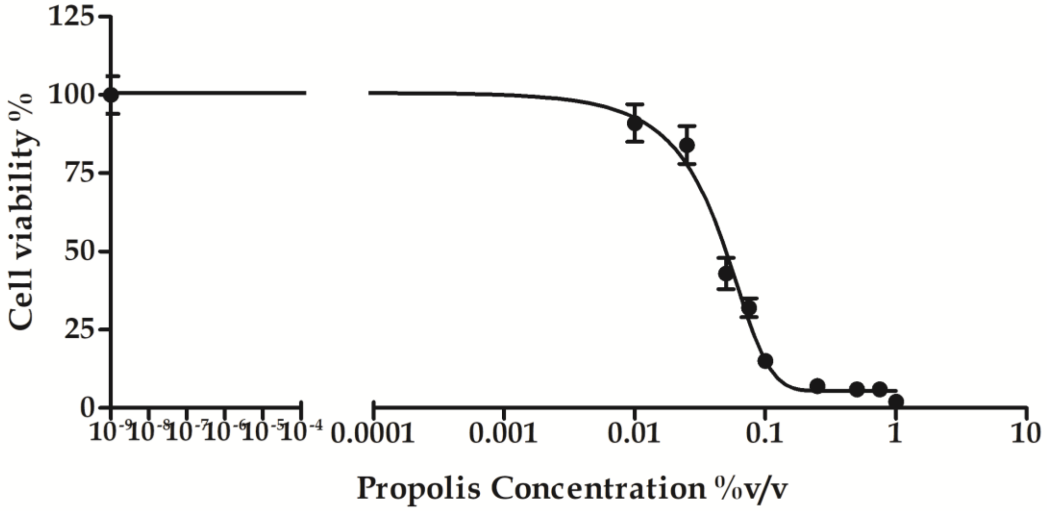

Dose-response curve showing cell viability (calcein-AM assay) after propolis treatment (propolis concentration %v/v)
